# *Listeria ivanovii* Infection in Mice: Restricted to the Liver and Lung with Limited Replication in the Spleen

**DOI:** 10.3389/fmicb.2016.00790

**Published:** 2016-06-07

**Authors:** Mengying Zhou, Mingjuan Jiang, Chenyan Ren, Sijing Liu, Qikang Pu, Howard Goldfine, Hao Shen, Chuan Wang

**Affiliations:** ^1^Food Safety Monitoring and Risk Assessment Key Laboratory of Sichuan Province, Department of Public Health Laboratory Sciences, West China School of Public Health, Sichuan UniversityChengdu, China; ^2^Department of Microbiology, Perelman School of Medicine, University of PennsylvaniaPhiladelphia, PA, USA

**Keywords:** *Listeria ivanovii*, *Listeria monocytogenes*, pathogenicity, immunogenicity, liver, lung

## Abstract

*Listeria monocytogenes* (LM) vectors have shown much promise in delivery of viral and tumor antigens for the development of vaccines. *L. ivanovii* (LI) is a closely related bacterium with a similar intracellular life cycle that may offer advantages over LM because it is not a human pathogen, but can infect other animal species. Recent studies show that recombinant LI expressing *Mycobacterium tuberculosis* antigens is effective in inducing protective immunity in mouse models, demonstrating the potential of LI as a live vaccine vector. However, a key barrier in the development of LI into a live vaccine vector is that its pathogenic and immunogenic characteristics have yet to be fully understood. Therefore, in this research, C57BL/6J mice were inoculated with LM or LI intravenously or intranasally, and bacterial loads, histopathologic changes, and cytokine production were determined at indicated days post inoculation. Results showed that after intravenous infection with LM or LI, bacteria were found proliferating in the liver, spleen, and lung. However, LI could only reach a heavy burden in the liver and its ability to multiply and to resist host immunity seemed limited in the spleen and lung. After intranasal inoculation with LI, bacteria were mainly localized in the lung and failed to infect liver or spleen, while LM could. In organs with heavy LI burden, lesions were isolated, localized and densely packed, compared to lesions caused by LM, which were invasive. In the liver of intravenously inoculated mice and lung of intranasally inoculate mice, LI was able to elicit comparable cytokine production with LM and cause less severe histopathologic damages, and thus could be considered as a vector for treating or preventing hepatic or pulmonary diseases.

## Introduction

*Listeria monocytogenes* (LM) is a Gram-positive, food-borne pathogen responsible for human and animal listeriosis, characterized by severe gastroenteritis, central nervous system infections, and mother-to-child infections, with an overall mortality rate of 30% (Roberts and Wiedmann, 2003). LM is capable of multiplying in the cytoplasm of phagocytic and non-phagocytic cells and directly infecting adjacent cells, guaranteeing efficient antigen processing and presentation, which thereby enables it to elicit robust innate and antigen-specific cellular immunity (Stavru et al., 2011). Consequently, LM is now being used as both a model to investigate host immune responses as well as a vaccine vector for T cell immunity (Singh and Wallecha, 2011; Stavru et al., 2011). Research into LM-based vaccines has made great progress in the last 20 years. Diverse antigen genes, such as genes of tumor related antigens and virus antigenic genes, have been genetically recombined into LM or attenuated strains, exhibiting desirable results *in vivo*, including tumor shrinkage, activated T cell immunity, and survival following higher challenging doses in mice (Mata et al., 2001; Brockstedt et al., 2004; Wood et al., 2011). So far, five LM-based vaccine candidates have been tested and are in various phases of clinical trials (Le et al., 2012).

In comparison, *L. ivanovii* (LI), the other pathogenic bacteria in the *Listeria* genus, is strictly limited to ruminant infection, except for extremely rare cases of infection in immunocompromised people (Dominguez-Bernal et al., 2006; Snapir et al., 2006; Guillet et al., 2010). Despite its different host tropism, LI is the bacterial species most similar to LM, sharing similar mechanisms for phagosome escape, intracellular mobility, reproduction, and cell-to-cell spreading (Vazquez-Boland et al., 2001b; Dominguez-Bernal et al., 2006). Therefore, LI could potentially be a promising vaccine vector even better than LM considering its lower pathogenicity in humans. Unfortunately, no research on LI-based vaccines has been reported until very recently, when two recombinant LI strains expressing *Mycobacterium tuberculosis* ESAT-6 or Ag85C protein were constructed and tested *in vivo* as tuberculosis vaccine candidates (Lin et al., 2015). Specific gamma interferon (IFN-γ) secretion of activated CD8 T cells was observed, which indicated the possibility of using LI as vaccine vector. However, the pathogenic and immunogenic features of LI and differences to LM remain poorly understood. Therefore, in order to correctly utilize LI as a novel live bacteria vaccine vector, it is important to conduct comprehensive studies such as the one detailed here.

In this study, C57BL/6J mice were inoculated intravenously (i.v.) or intranasally (i.n.), two effective routes of administration already established in C57BL/6J mice (Mayuko et al., 2002). Then, bacterial loads, histopathology and cytokine secretion kinetics along with infection course were systematically studied and compared between LM and LI infected mice.

## Materials and Methods

### Animals

Female C57BL/6J mice were purchased from Beijing HFK Bioscience Company (Beijing, China). Mice aged 6–8 weeks were used in this study. All mice were maintained under specific-pathogen-free conditions throughout the experiments at the Animal Center of School of Public Health at Sichuan University. Mouse experiments were performed according to the guidelines of the Animal Care and Use Committee of Sichuan University.

### Bacteria and Inoculum Preparation

LM 10403s strain and LI PAM55 strain were used in this study. All bacteria were stored in brain-heart infusion (BHI) broth containing 50% glycerol at -80°C. Bacteria were recovered in BHI broth from -80°C storage, and cultured in fresh BHI broth at 37°C and 200 rpm to mid-logarithmic phase (OD600≈0.4), then centrifuged at 4°C at 12000 *g* for 2 min, washed with normal saline, and then resuspended in normal saline. Titers of live bacteria were adjusted according to OD600 vs. bacteria concentration curves obtained in our earlier work (data not shown). Actual numbers of colony forming units (CFUs) of bacteria were verified by plating diluted inoculums on BHI agar plates.

### Mouse Infection and Sacrifice

One hundred and sixty-two mice were randomly distributed into four infection groups (36 mice in each) and one naive group (18 mice). Two infection groups of mice were i.v. infected with 5 × 10^4^ CFU LM or 5 × 10^5^ CFU LI in a volume of 100 μl. The other two infection groups of mice were anesthetized by diethyl ether, and then i.n. inoculated with 5 × 10^5^ CFU LM or 5 × 10^7^ CFU LI in 25 μl of normal saline. All infection doses were 10-fold lower than the 50% lethal doses determined previously (data not shown). Naive group of mice were untreated. At 1, 2, 3, 5, and 8 each day(s) post inoculation (dpi), six mice in each i.v. infection group were sacrificed for analysis of bacterial loads and cytokine titers in organs. At 1, 3, 5, 8, and 11 each dpi, six mice in each i.n. infection group were sacrificed for the same analysis. At 4 and 6 each dpi, six mice in naive group were sacrificed for the same analysis. Additionally, six mice in each group were sacrificed for histopathologic analysis at 3 dpi (i.v. group), 5 dpi (i.n. group), or 4 dpi (naive group).

### Determination of Bacteria Load in Organs from *Listeria*-Infected Mice

Liver, spleen, and lung were removed aseptically. Around 0.05 g tissue (the smallest lobe of liver, half spleen and one lobe of lung) was cut off and stored in liquid nitrogen for cytokine determinations. The rest of the tissue was weighed and homogenized in sterilized and pre-cooled phosphate buffer solution (PBS) containing 0.1% Triton-X100. Serial dilutions of homogenates were plated on BHI agar plates and cultured at 37°C. Colonies were counted after 24 h for LM or after 48 h for LI.

### Histopathologic Analysis of Tissue from *Listeria*-Infected Mice

Liver, spleen, and lung were fixed in 10% buffered formalin, dehydrated and embedded in paraffin. Sections of 5 μm were cut, stained with hematoxylin-eosin and examined under 100 × magnification.

### Preparation of Organ Extracts and Luminex Measurements of Cytokines

Frozen tissue was removed from liquid nitrogen, and ground in a cold mortar. Lysis buffer (50 mmol/L Tris-HCl pH7.4, 150 mmol/L NaCl, 1mmol/L EDTA, 1% Triton X-100, 1mmol/L phenylmethanesulfonyl fluoride, 5 μg/ml aprotinin, 5 μg/ml leupeptin) 100 μl, was added to lyse the tissue. Samples were incubated on ice for 10 min and vortexed for 2 min, repeating twice. Samples were then centrifuged at 4°C and 16000 *g* for 10 min, and the supernatant was collected. Total protein concentration of each sample was measured using BCA Protein Assay kit (Beyotime, Shanghai, China) and was diluted to 10 mg/ml with PBS. All samples were stored at -80°C. Cytokine measurements were performed with a Luminex^®^100^TM^ instrument using a ProcartaPlex Mouse Essential Th1/Th2 Cytokines Panel (eBioscience, San Diego, CA, USA). All procedures of cytokine determination were based on the manufacturer’s instructions.

### Statistical Analysis

Differences of daily weight between LM and LI infection mouse models were analyzed by *t*-test. Bacterial loads and cytokine titers in organs were compared by using the Mann–Whitney U non-parametric test. In all experiments, *P* < 0.05 was considered significant.

## Results

### Kinetics of LM and LI Growth in Organs from Intravenous and Intranasal Infection Models

**FIGURE 1:**
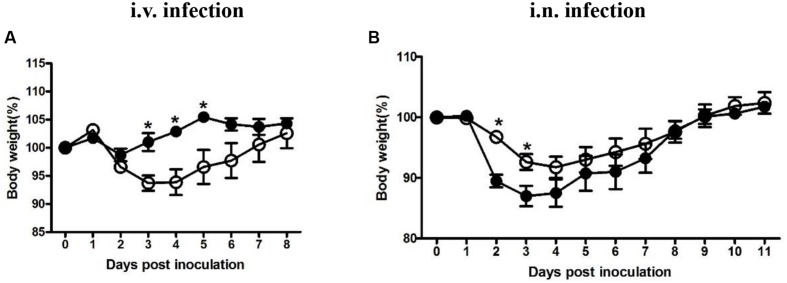
**Weight changing percentages after intravenous or intranasal inoculation with LM and LI.** Mice were i.v. inoculated with 5 × 10^4^ CFU LM or 5 × 10^5^ CFU LI **(A)**, or i.n. inoculated with 5 × 10^5^ CFU LM or 5 × 10^7^ CFU LI **(B)**. Weight changing percentages were calculated and compared between LM (●) and LI (aaa) at the same time point by the method of *t* test (^∗^*P* < 0.05). Each point represents mean ± standard error of the mean of six mice per group.

Four groups were either inoculated with 5 × 10^4^ CFU LM (i.v.), 5 × 10^5^ CFU LI (i.v.), 5 × 10^5^ CFU LM (i.n.), or 5 × 10^7^ CFU LI (i.n.). Each dose was 10-fold lower than the 50% lethal dose determined previously (data not shown). As an additional parameter of measurement during the course of infection, percentages of weight change after infection were taken daily (**Figure 1**). In intravenous infection model, mice infected either *Listeria* species started to lose weight at 1 dpi and then recovered gradually (**Figure 1A**). However, mice infected with LM reached maximum weight loss (6% of original weight) at 3 and 4 dpi and returned to original weight at 7 dpi, while mice infected with LI showed only slight weight loss (**Figure 1A**). After intranasal infection, mice infected with either LM or LI started to lose weight over 1 to 4 dpi and recovered to original weight at 9 dpi, but more weight loss was induced by LI infection (**Figure 1B**). The maximum weight loss for mice intranasally infected with LM was 8% of original weight while that for mice infected with LI was 13% of original weight (**Figure 1B**).

**FIGURE 2:**
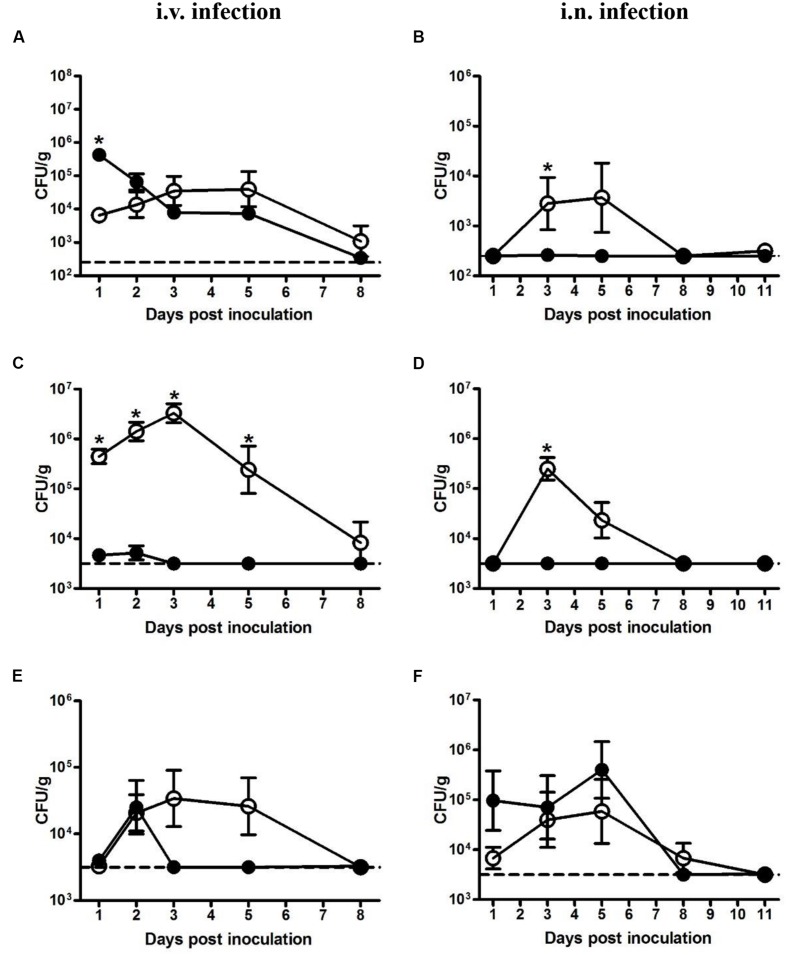
**Kinetics of LM and LI loads in liver, spleen, and lung from intravenous and intranasal infection models.** Mice were i.v. inoculated with 5 × 10^4^ CFU LM or 5 × 10^5^ CFU LI, and bacterial loads in the liver **(A)**, spleen **(C),** and lung **(E)** were determined. Mice were i.n. inoculated with 5 × 10^5^ CFU LM or 5 × 10^7^ CFU LI, and bacterial loads in the liver **(B)**, spleen **(D),** and lung **(F)** were determined. The number of LM (●) and LI (aaa) in certain organ at indicated time points were compared by the method of the Mann–Whitney U non-parametric test (^∗^*P* < 0.05) at the same time point. Each point represents the mean ± standard error of the mean for a group of six mice. The dotted lines represent the detection limits in each experiment.

Bacterial loads in the liver, spleen, and lung were determined at indicated time points. In mice intravenously inoculated with 5 × 10^4^ CFU LM, the bacterial load in the liver, though significantly less than that of LI at 1 dpi, kept increasing and reached maximum load of 4 × 10^4^ CFU/g at 3 dpi (**Figure 2A**). By contrast, in mice intravenously inoculated with 5 × 10^5^ CFU LI, about 88% of the bacteria invaded liver, causing the bacteria load in liver to reach 4 × 10^5^ CFU per g then decreased gradually (**Figure 2A**). However, in the spleen after intravenous infection, LM showed notable and prolonged growth (**Figure 2C**). In mice intravenously inoculated with 5 × 10^5^ CFU LI, the maximum load was 5 × 10^3^ CFU per g spleen, while for mice inoculated with a smaller dose of LM, the maximum value was 3 × 10^6^ CFU per g spleen (**Figure 2C**). Additionally, the load of LI in the spleen decreased below the detection limit after 3 dpi, but LM were still detectable at 8 dpi, showing that LI was cleared rapidly in host spleen (**Figure 2C**). In the lung after i.v. infection, though both LM and LI could reach a maximum load around 3 × 10^4^ CFU per g lung, the former could resist host immunity and persist in the lung for over 5 days while the latter only persisted for 3 days, again showing its weak ability to maintain infection (**Figure 2E**).

In the liver from intranasal infection model, LM proliferated after 1 dpi, reached a peak value of 4 × 10^3^ CFU per g liver at 5 dpi, and was undetectable at 8 dpi. In stark contrast, LI were hardly detectable for the duration of the experiment even though the dose was 100-fold higher than LM (**Figure 2B**). The same situation was also observed in the spleen, where levels of LM peaked at 3 dpi (10^5^ CFU per g spleen), while LI remained at undetectable level for the duration of the entire experiment (**Figure 2D**). However, a difference was observed in the lung following intranasal inoculation. The bacterial load of LM increased after 1 dpi, peaked at 5 dpi, and gradually declined to undetectable levels at 11 dpi, presenting a distinct proliferative process (**Figure 2F**). In contrast, the load of LI maintained around 10^5^ CFU per g lung for the first 5 days and sharply dropped below the detection limit at 8 dpi (**Figure 2F**). Though the maximum load of LI (4 × 10^5^ CFU per g lung) in the lung were slightly higher than that of LM (6 × 10^4^ CFU per g lung), the inoculation dose of LI was 100-fold higher, indicating that the LI could not resist host immunity and multiply as well as LM could in the lung. Taken together, after intranasal inoculation LI was mainly localized in the lung and could barely invade liver or spleen, while LM could multiply in these three organs, again demonstrating LI is less pathogenic.

### Histopathologic Analysis of Organs Infected with LM or LI from Intravenous and Intranasal Infection Models

**FIGURE 3:**
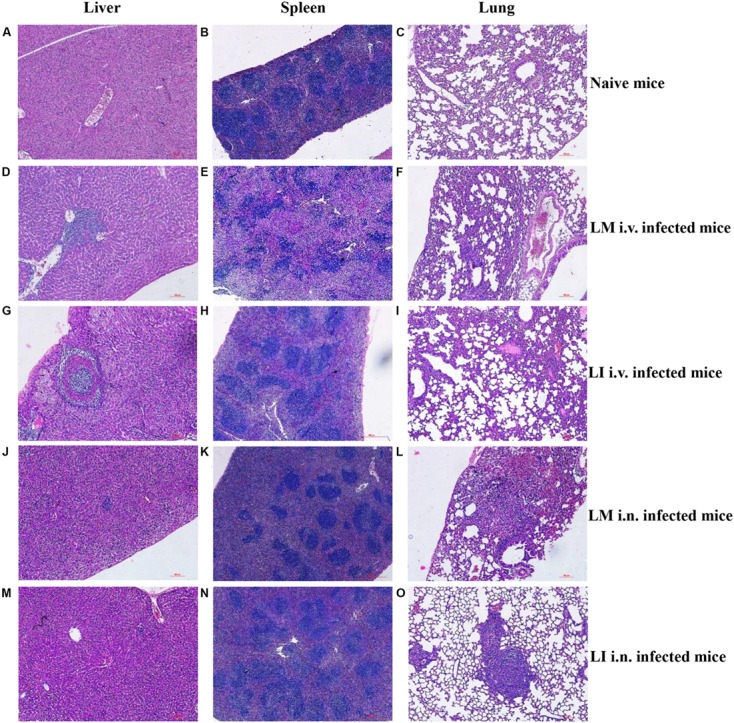
**Histopathologic analysis of liver, spleen, and lung from LM and LI intravenous and intranasal infection models.** Mice were, respectively, inoculated with 5 × 10^4^ CFU LM (i.v.), 5 × 10^5^ CFU LI (i.v.), 5 × 10^5^ CFU LM (i.n.) or 5 × 10^7^ CFU LI, along with a group of naive mice. I.v. infected mice were sacrificed at 3 dpi, i.n. infected mice were sacrificed at 5 dpi, and naive mice were sacrificed at 4 dpi. Histologic sections of liver, spleen, and lung were made and stained with hematoxylin-eosin. Liver and lung sections were observed under a 40× microscope and spleen sections were observed under a 10× microscope. The images presented are representatives of changes seen in naïve mice **(A–C)**, LM i.v. **(D–F)**, LI i.v. **(G–I)**, LM i.n. **(J–L),** and LI i.n. **(M–O)**.

To study and compare tissue damage in host organs caused by LM or LI, mice that were intravenously infected were sacrificed at 3 dpi, mice that were intranasally infected were sacrificed at 5 dpi, and naive mice were sacrificed at 4 dpi. Histologic sections of liver, spleen, and lung samples were then made and analyzed (**Figure 3**). In the livers from intravenous infection model, lesions caused by LM were numerous and mainly located near vessels (**Figure 3D**), while lesions caused by LI were fewer but larger in size, featuring layers of necrotic hepatocytes and lymphocytes surrounding each other (**Figure 3G**). In the spleen from intravenous infection model, however, histopathology was consistent with bacteria load. LM infection led to severe necrosis which expanded to entirely efface the normal splenic architecture (**Figure 3E**), while no substantial changes were observed in LI infected spleens (**Figure 3H**). In the lung after i.v. infection, collapsed alveoli accompanied with lymphocytes were observed, along with more numerous and severe lesions for LM-infected mice (**Figure 3F**) than for LI-infected mice (**Figure 3I**). Compared to the intravenous infection model, both LM and LI inoculated intranasally presented smaller and fewer hepatic lesions and hardly observed splenic necrosis (**Figures 3J,K,M,N**). However, tissue damage of the lungs was severe and differed between mice infected with the two bacterial strains. Following intranasal infection with LM, pulmonary necrosis along with inflammatory infiltration expanded to a large area (**Figure 3L**). In contrast, lung lesions caused by LI were isolated and densely packed in a pattern similar to LI intravenously infected liver, where the bacterial burden was also heavy (**Figure 3O**). Histopathological results were consistent with bacterial loads, in that severe histopathologic changes were observed in tissues with high bacterial loads. However, lesions caused by LI were found isolated and densely packed which indicated the ability of LI to expand infection foci might be limited.

### Kinetics of Cytokine Production in Organs Infected with LM or LI from Intravenous and Intranasal Infection Models

**FIGURE 4:**
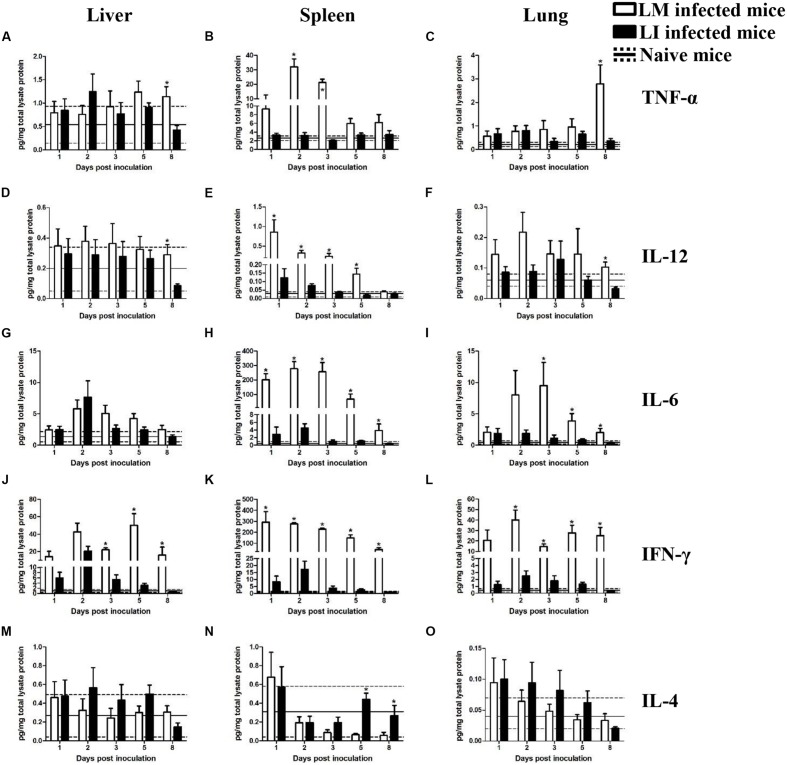
**Kinetics of cytokine production in organs from LM and LI intravenous infection models.** Mice were, respectively, inoculated with 5 × 10^4^ CFU LM (white bar) or 5 × 10^5^ CFU LI (black bar), and one group of naive mice were set (solid line represents average value and the dotted line represents average value plus/minus standard deviation). Titers of TNF-α **(A–C)**, IL-12 **(D–F)**, IL-6 **(G–I)**, IFN-γ **(J–L),** and IL-4 **(M–O)** in the liver, spleen, and lung were determined at 1, 2, 3, 5, and 8 dpi for *Listeria* infected mice or at 4 dpi for naive mice. The differences of titers at each test day were compared between LM and LI by the method of the Mann–Whitney U non-parametric test (^∗^*P* < 0.05). Each bar represents the mean ± standard error of the mean for a group of six mice.

**FIGURE 5:**
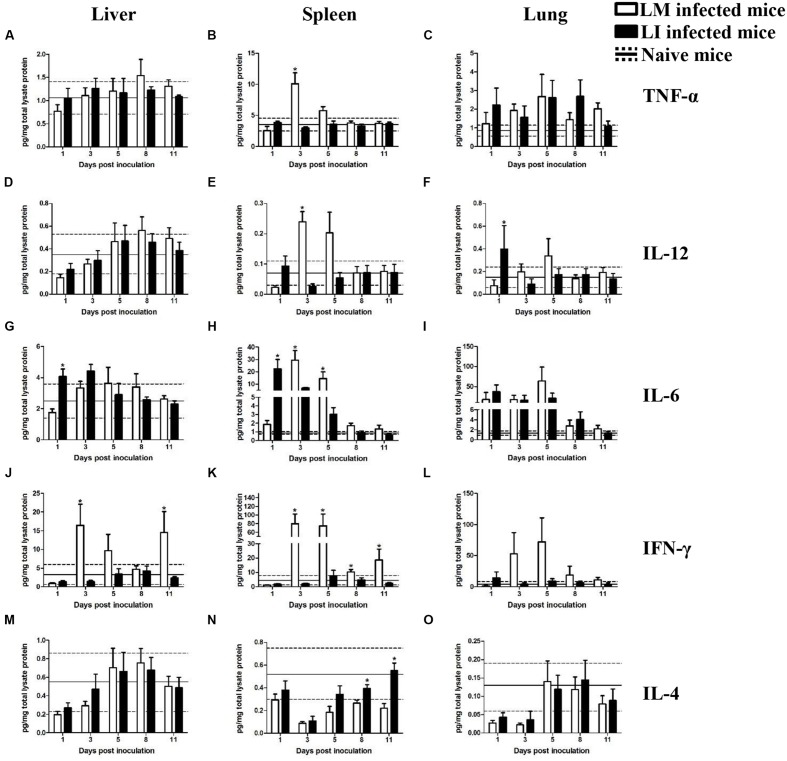
**Kinetics of cytokines production in organs from LM and LI intranasal infection models.** Mice were, respectively, inoculated with 5 × 10^5^ CFU LM (white bar) or 5 × 10^7^ CFU LI (black bar), and one group of naive mice were set (solid line represents average value and the dotted line represents average value plus/minus standard deviation). Titers of TNF-α **(A–C)**, IL-12 **(D–F)**, IL-6 **(G–I)**, IFN-γ **(J–L),** and IL-4 **(M–O)** in the liver, spleen, and lung were determined at 1, 3, 5, 8, and 11 dpi for *Listeria* infected mice or at 6 dpi for naive mice. The differences of titers at each test day were compared between LM and LI by the method of the Mann–Whitney U non-parametric test (^∗^*P* < 0.05). Each bar represents the mean ± standard error of the mean for a group of six mice.

To investigate and compare inflammatory responses associated with LM and LI infection via intravenous (**Figure 4**) or intranasal (**Figure 5**) infection route, four groups of mice were inoculated accordingly. One group of mice was left uninfected as a naive control group. At indicated time points, Luminex beads assay was performed to determine cytokine titers in the liver, spleen, and lung, including tumor necrosis factor alpha (TNF-α), interleukin-12 (IL-12), interleukin-6 (IL-6), interleukin-4 (IL-4), and IFN-γ. The kinetics of cytokine production in mice infected with LM or LI differed by organs and inoculation routes.

#### Intravenous Infection Model

##### Liver

In the liver after intravenous injection, cytokines titers in LI inoculated mice were generally at comparable levels to titers in LM inoculated mice (**Figure 4**). TNF-α and IL-12 titers showed no substantial differences between mice infected with LM and LI at 1, 2, 3, and 5 dpi, except at 8 dpi (**Figures 4A,D**). IL-6 production was increased and reached a peak titer at 2 dpi after both *Listeria* infections and no difference of daily IL-6 production was found between LM and LI-infected mice (**Figure 4G**). IFN-γ titers in the liver were notably increased at 2 dpi in both LM and LI infected mice, but IFN-γ titer in LI infected mice decreased after 3 dpi while that in LM infected mice remain at a high level (**Figure 4J**). IL-4, an antagonistic factor in anti-listeriosis response, showed no differences between mice inoculated with the two *Listeria* strains within the 5 experimental days (**Figure 4M**).

##### Spleen

In the spleen following intravenous infection, however, cytokines production induced by LI were at significantly lower levels when compared with LM. For example, TNF-α titer for LM infected mice was significantly elevated (32.0 and 21.3 pg/mg total lysate protein) at 2 and 3 dpi, respectively, while the titer for LI for the same dpis were around 3.0 pg/mg total lysate protein, not significantly higher than it of naive mice (**Figure 4B**). The titers of IL-12 were increased after infection with either *Listeria* species, but the titer of LM infected mice was much higher (**Figure 4E**). Moreover, levels of both IL-6 and IFN-γ induced by LI were lower than those induced by LM at each experimental day, but they were still higher than those of naive mice and reached peak values of 4.5 pg/mg total lysate protein IL-6 and 17.3 pg/mg total lysate protein IFN-γ at 2 dpi (**Figures 4H,K**). IL-4 titer in the spleen kept declining during the course of LM infection, whereas titers for LI infection declined first and then returned to the level of naive control group since 5 dpi (**Figure 4N**).

##### Lung

No substantial differences in TNF-α, IL-12, and IL-4 secretion in the lung were found between LM and LI infected mice, but pulmonary IL-6 and IFN-γ induced by LM were significantly higher. No difference in titer of TNF-α was observed between the two bacteria in intravenously infected mice until 8 dpi, when a high titer of TNF-α (2.8 pg/mg total lysate protein) was found in the LM-infected mice (**Figure 4C**). Similarly, the difference of IL-12 titer between LM and LI occurred only at 8 dpi with IL-12 titer as low as 0.03 pg/mg total lysate protein in LI-infected mice (**Figure 4F**). IL-6 production in the lung after LM infection showed an obvious increase up to 9.5 pg/mg total lysate protein at 3 dpi, while the production after LI infection was barely increased (**Figure 4I**). Titers of pulmonary IFN-γ in LM infected mice was at least five times higher than that in LI infected mice (**Figure 4L**). No differences were found in IL-4 secretion in the two *Listeria* infected mice (**Figure 4O**).

#### Intranasal Infection Model

##### Liver

Cytokine titers in the liver and spleen were generally lower in the intranasal infection model than in the intravenous infection model, however, cytokine production in the lung were higher after i.n. infection than after i.v. infection, a finding consistent with bacterial burdens (**Figure 5**). In the liver from intranasal infection model, production of cytokines, except IFN-γ, were not notably promoted after infection with either *Listeria*. Kinetics of TNF-α, IL-12, and IL-4 induced by the two bacteria were the same (**Figures 5A,D,M**). IL-6 titer only differed at 1 dpi with 1.8 pg/mg total lysate protein for the LM infected mice and 4.1 pg/mg total lysate protein for the LI infected mice (**Figure 5G**). IFN-γ production, however, were significantly different between LM and LI i.n. infected mice. Hepatic IFN-γ in LI inoculated mice slightly changed within the range of naïve mice, while LM induced IFN-γ highly secreted in the liver, 16.5 pg/mg total lysate protein at 3 dpi and 14.5 pg/mg total lysate protein at 11 dpi (**Figure 5J**).

##### Spleen

In the spleen from intranasal infection model, cytokine levels in LM infected mice were notably increased at 3 dpi while changes of cytokine levels in LI-infected mice were less significant. Titers of TNF-α and IL-12 were obviously increased to 10.1 pg/mg total lysate protein and 0.2 pg/mg total lysate protein at the third day after infection with LM while production of TNF-α and IL-12 barely changed after infection with LI (**Figures 5B,E**). The peak for IL-6 secretion occurred at 1 dpi with a titer of 22.5 pg/mg total lysate protein in LI infected mice, but in LM infected mice, the IL-6 peak occurred at 3 dpi with a titer of 29.3 pg/mg total lysate protein (**Figure 5H**). IFN-γ production was significantly higher following LM intranasal infection at 3, 5, 8, and 11 dpi (**Figure 5K**). Titers of IL-4 decreased after infection with both *Listeria* but it recovered earlier in LI infected mice (**Figure 5N**).

##### Lung

In the lung after intranasal infection, where the bacteria were mainly focused, cytokine titers were generally higher than those in the lung from the intravenous infection model, but fewer differences were found between mice infected with LM and LI. TNF-α, IL-6, and IL-4 production separately shared similar trends and levels in mice infected with LM or LI (**Figures 5C,I,O**). The peak of IL-12 was at 1 dpi in LI infected mice but occurred at 3 dpi in LM infected mice (**Figure 5F**). The titer of IFN-γ was higher in LM intranasally infected lung at 3, 5, 8, and 11 dpi, and the biggest difference presented at 5 dpi with 72.1 pg/mg total lysate protein in LM infected mice but only 9.4 pg/mg total lysate protein in LI infected mice (**Figure 5L**). However, the difference was not statistically significant.

## Discussion

In this study pathogenic and immunologic outcomes of LI intravenous or intranasal infections in C57BL/6J mice were investigated in comparison with LM. In intravenous infection model, bacteria were delivered into the bloodstream and directly distributed to organs. In the spleen and lung, LI showed reduced pathogenicity compared with LM, as demonstrated by low and transient bacterial loads and minor histopathologic changes. In the liver, however, LI could rapidly reach a high burden, caused notable pathogenic damages and then cleared by host gradually. It indicated that LI in blood were mainly distributed to liver, which, to our knowledge, is related to a protein called Internalin B (InlB). In LM, InlB is found to trigger the entry of LM into hepatocytes by interacting with a hepatocyte growth factor receptor and two other cellular components (Bierne and Cossart, 2002). LM mutant lacking the gene encoding InlB (*inlB*) displays a marked decrease in its capability to proliferate within mouse hepatocytes *in vivo* and *in vitro* (Gregory et al., 1997). The existence of InlB for LI has been proved by gene sequencing. Two genes named *i-inlB1* and *i-inlB2* were found to be genetically similar to *inlB* of LM, and were thought to code for an InlB ortholog in LI (Dominguez-Bernal et al., 2006). Comparing their amino acid sequences, both i-InlB1 and i-InlB2 share a similar architecture with InlB for the internalin domain and bacterial-surface anchoring domain, indicating that i-InlB1 and i-InlB2 may mediate the interaction between LI and hepatocytes. However, detailed differences in structure and function *in vivo* among these three internalins and how i-InlB1 and i-InlB2 interact hepatocytes are still wanted.

Another finding in intravenous infection model is that LI failed to keep multiplying as long as LM did and were cleared by host more rapidly, especially in the spleen and lung. After analyzing typical histopathologic changes of LI (**Figures 3G,O**), it was found that LI infection foci was isolated and densely packed while LM infection foci was invasive, indicating that, compared with LM, the abilities of LI to multiply intracellularly and/or to invade neighboring cells are limited. It has been proved that LI is not as capable of multiplying in splenic cells as LM *in vitro* (Kimoto et al., 2003). This flaw could be related to LI’s poor ability to lyze phagosome, since LM mutant unable to effectively disrupt phagosome and escape into cytoplasma is soon cleared (Gaillard et al., 1986). Phagosome lysis is initiated by three LM virulence determinants: listeriolysin O (LLO) and two phospholipases (PlcA and PlcB; Vazquez-Boland et al., 2001b). These three molecules are also found in LI. Ivanolysin O (ILO) is the homolog of LLO in LI. A LM mutant expressing ILO instead of its own LLO failed to proliferate in the spleen, inducing a high-level of IFN-γ, as well as generating robust protective immunity (Frehel et al., 2003; Hideki et al., 2007), which indicates the weak ILO may be responsible for deficient intracellular multiplication of LI. PlcA and PlcB of LI are only verified genetically, and no details about their structure and function are available (Vazquez-Boland et al., 2001a). After escaping from the ruptured phagosome, LM replicates in the cytoplasm and expresses the surface molecule ActA which provides the bacteria with actin-based motility and propels it through membrane protrusions into neighboring cells to expend infection to a large area (Vazquez-Boland et al., 2001b). In LI, the ActA-like protein is called i-ActA and can restore mobility of LM *actA* deletion mutant strain *in vitro* (Gouin et al., 1995). However, detailed differences between i-ActA and ActA in the process of cell-to-cell infection remain unclear. So far, major virulence factors of LM has been genetically recognized in LI (Vazquez-Boland et al., 2001a). Similarities in genes between these homologs imply similar functions, but it is the differences that help to explain different behavior *in vivo* between LM and LI. More information is needed to make reliable conclusions.

In this research, immunologic features of LI in different organs after different inoculation were also conducted in comparison with LM. Five cytokines were adopted to reflect different aspects of host anti-*Listeria* immune response, due to sufficient data of host immunity to LM. TNF-α, IL-6, and IL-12 are secreted by LM infected macrophages to recruit and activate neutrophils and NK cells, which are both capable of killing infected cells (Harty et al., 1996; Mocci et al., 1997). IFN-γ is mainly secreted by activated NK cells to enhance bactericidal actions inside infected macrophages and promote expression of signal molecules on surface to further amplify immune stimulation (Pamer, 2004). Apart from innate immune response, T cell-mediated immune response also takes part in host immunity to first time infection of LM. IL-6 and IL-12 play a role in promoting Th0 cells to develop into Th1 cells, while IL-4 suppresses the development (Kaufman et al., 1997; Mocci et al., 1997). In this research, though the magnitude and duration of each cytokine differed between the two *Listeria*, the trends along with infection are same, which are increase of TNF-α, IL-6, IL-12, and IFN-γ and decrease of IL-4. The same secretion trend of cytokines implies host immune response to LI is similar with it to LM. However, in most cases, cytokine response to LM was stronger than that to LI, which was likely due to different behavior in tissues between LM and LI. In infected tissue, LM increased in number exponentially while LI was either cleared or not growing, and thus LI did not induce cytokine response as robust as LM did. This phenomenon, on the one hand, proves that LI is less virulent, and on the other hand indicates that LI, as a vaccine vector, needs optimization for stronger immunogenicity.

Nevertheless, cytokine secretion induced by LI infection was at comparable level of LM infectoin in these two situations: in the liver from i.v. inoculated mice and in the lung from i.n. inocualted mice. Titers of TNF-α, IL-6, IL-12, and IL-4 after infection changed at same level between LI and LM infections. IFN-γ in the liver after i.v. infection dropped earlier in LI infected mice than LM infected mice because LI could be cleared rapidly. IFN-γ in the lung after i.n. infection seemed higher in LM infected mice at 3 and 5 dpi, but the difference was not statistically significant due to individual differences. In general, LI could mainly infect mouse liver after i.v. inoculation and specifically infect mouse lung after i.n. inoculation. In these two situations, LI could induce immune response not significantly weaker than LM, cause less sever tissue damages and could be controlled by host more easily. Therefore, speculation is that LI, after being genetically improved for better immunogenicity, could be used as vaccine vector specific for hepatic or pulmonary disease, especially for immunocompromised people.

## Author Contributions

MZ, CW, and HS designed the study. MZ, MJ, CR, SL, and QP performed the experimental work. MZ analyzed the data and prepared the manuscript. CW, HG, and HS contributed to the final manuscript.

## Conflict of Interest Statement

The authors declare that the research was conducted in the absence of any commercial or financial relationships that could be construed as a potential conflict of interest.
